# Differential MicroRNA Profiling in a Cellular Hypoxia Reoxygenation Model upon Posthypoxic Propofol Treatment Reveals Alterations in Autophagy Signaling Network

**DOI:** 10.1155/2013/378484

**Published:** 2013-12-22

**Authors:** Zhuo Chen, Zhe Hu, Zhiqi Lu, Shuyun Cai, Xiaoxia Gu, Haixia Zhuang, Zhihua Ruan, Zhengyuan Xia, Michael G. Irwin, Du Feng, Liangqing Zhang

**Affiliations:** ^1^Department of Anesthesiology, Affiliated Hospital of Guangdong Medical College, Zhanjiang 524001, China; ^2^Anesthesiology Research Laboratory, Department of Anesthesiology, University of Hong Kong, Hong Kong; ^3^Key Laboratory of Age-Associated Cardiac-Cerebral Vascular Disease of Guangdong Province, Department of Neurology, Affiliated Hospital of Guangdong Medical College, Zhanjiang 524001, China

## Abstract

Recent studies indicate that propofol may protect cells via suppressing autophagic cell death caused by excessive reactive oxygen species induced by hypoxia reoxygenation (H/R). It is established that gene expression patterns including autophagy-related genes changed significantly during the process of H/R in the presence or absence of propofol posthypoxia treatment (P-PostH). The reasons for such differences, however, remain largely unknown. MicroRNAs provide a novel mechanism for gene regulation. In the present study, we systematically analyzed the alterations in microRNA expression using human umbilical vein endothelial cells (HUVECs) subjected to H/R in the presence or absence of posthypoxic propofol treatment. Genome-wide profiling of microRNAs was then conducted using microRNA microarray. Fourteen miRNAs are differentially expressed and six of them were validated by the quantitative real-time PCR (Q-PCR) of which three were substantially increased, whereas one was decreased. To gain an unbiased global perspective on subsequent regulation by altered miRNAs, predicted targets of ten miRNAs were analyzed using the Gene Ontology (GO) analysis to build signaling networks. Interestingly, six of the identified microRNAs are known to target autophagy-related genes. In conclusion, our results revealed that different miRNA expression patterns are induced by propofol posthypoxia treatment in H/R and the alterations in miRNA expression patterns are implicated in regulating distinctive autophagy-related gene expression.

## 1. Introduction

As a result of increased oxidative stress, reperfusion of ischemic tissues or cells leads to a systemic inflammatory response which in turn may cause widespread microvascular dysfunction and tissue/cell injury [1–3]. Studies have shown that the vascular endothelium is a crucial site that is affected by ischemia/reperfusion (I/R) injury [4–6]. Due to the instability of the current overall animal model of I/R injury which is largely affected by multifaceted factors *in vivo* and *in vitro*, animal models thus cannot reflect the exact protective mechanisms of drugs on the tissue/cell damage. *In vitro* cultured endothelial cells, however, are simple and controllable system that can create useful I/R injury model [7, 8].

Propofol is a widely used intravenous anesthetic with antioxidant capacity. It shows protective roles on the hydrogen peroxide (H_2_O_2_)-induced apoptosis in cardiac cells and myocardial ischemia and reperfusion (I/R) injury in rats [9, 10]. Recent evidences have shown that propofol may suppress the I/R activated autophagic cell death through affecting the expressions of autophagy-related genes [11–14]. But the mechanisms of the protective effects of propofol on HUVECs I/R injury have not been well studied.

MicroRNAs (miRNAs) are an evolutionarily conserved family of short noncoding RNAs, that negatively regulate genes in a cell via degradation or translation inhibition of their target mRNAs [15]. In recent years, many associations between disease mechanisms and specific miRNAs have been identified and confirmed using large-scale microarrary profiling and genetic approaches [16]. Studies provide an overview of the role of miRNAs in the development of I/R injury in the heart and kidney [17]. However, miRNAs that are associated with the protective effect of propofol on I/R injury remain largely unknown.

In the present study, we constructed an *in vitro* cellular hypoxia/reperfusion (H/R) model and found that propofol effectively reduced H/R injury. We performed systemic analysis of the alterations in miRNA expression using miRNA microarray in human umbilical vein endothelial cells (HUVECs) treated with H/R in the presence or absence of propofol posthypoxia treatment. Fourteen miRNAs were shown to be differentially expressed of which eight were substantially increased, whereas six were decreased and six of them were picked and further validated by qRT-PCR. Then, the Gene Ontology (GO) analysis was conducted to build signaling networks of predicted targets of ten miRNAs. Interestingly, six of the identified microRNAs are found to target autophagy-related genes. Our results revealed that different autophagy-associated miRNA expression patterns are induced by propofol posthypoxia treatment in H/R, which implicates that differential autophagy-related gene-expression regulated by miRNAs may play a role in propofol posthypoxia treatment in H/R.

## 2. Results

### 2.1. Protective Effects of Propofol Posthypoxia Treatment against H/R Injury on Cell Viability

**Figure 1:**
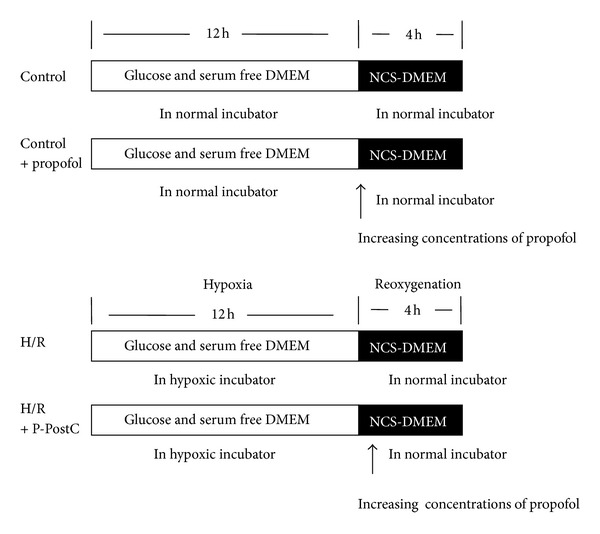
Build the H/R and P-PostH model. The culture media were replaced by glucose and serum free DMEM balanced in normal incubator in 30 minutes, and these HUVEC cells were then placed in hypoxic conditions which were created by a small enclosed humidified plexiglass chamber filled with 94% N_2_, 5% CO_2_, and 1% O_2_ at 37°C for 12 h. After hypoxia, the medium was immediately washed off, and the HUVECs were returned to the maintenance medium (NCS-DMEM) in normal incubator for 4 h. At the same time, prepared propofol was added to the medium to different concentrations (25 *μ*mol/L–150 *μ*mol/L).

**Figure 2:**
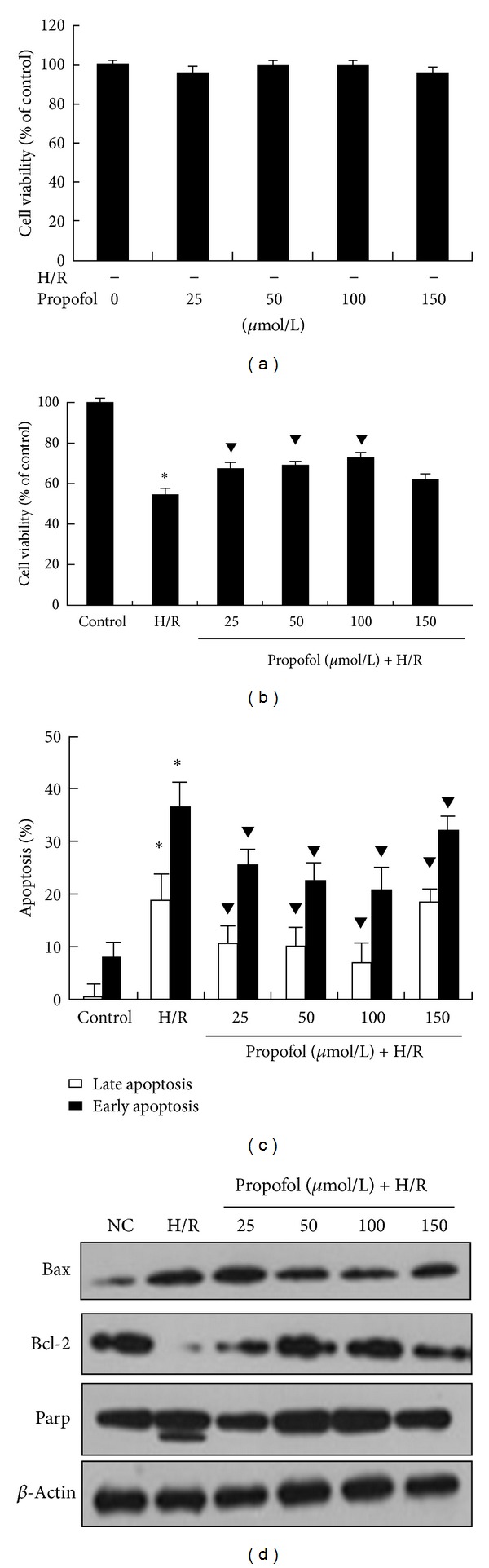
Propofol increased the viability and reduced the apoptosis of the H/R induced injury in HUVECs. (a) The normal HUVECs were treated with different concentrations (0–150 *μ*mol/L) of propofol for 4 h. (b) The cells were postconditioned with increasing concentrations of propofol (0–150 *μ*mol/L) after 12 h of hypoxia and 4 h of reperfusion. Cell viability was determined by CCK-8 assay, as previously described. Values are represented as the percentage of viable cells; vehicle-treated cells were considered as 100% viable. The data represented are mean percentage of viable cells + SD of three independent experiments. *P < 0.001, compared with control group, ^*▼*^ *P* < 0.05, compared with the H/R group. (c) Detection of apoptosis with Annexin V-FITC and propidium iodide staining. Every group of cells with Annexin V and propidium iodide staining was measured by flow cytometry. Histogram representing the percentage of early apoptotic cells and late apoptotic cells. The data represent the mean + SD of three independent experiments. *P < 0.01, compared with the control group, and ^*▼*^ *P* < 0.05 with H/R group. (d) Expression of apoptosis-related proteins in normal group, H/R injury group, and propofol posthypoxia treatment groups. Data are representative WB from 3 independent experiments (*n* = 2).

The construction of the cellular H/R P-PostH model is shown in Figure 1. The cells in the control group were considered 100% viable. As shown in Figure 2(a), there was no marked alteration in propofol-treated cells compared with control cells under normal conditions. CCK-8 assay showed that propofol treatment for 4 h at concentrations up to 150 *μ*mol/L did not influence cell viability (*P* > 0.05). Exposure of cells to H/R resulted in reduction of cell viability (Figure 2(b)). The viabilities of cells in the H/R groups were 55.9 ± 1.8% (*P* < 0.01 versus control). In all the groups of H/R+P-PostH, the viability of cells increased compared with the H/R only group (*P* < 0.05) except 150 *μ*mol/L (68.1 ± 5.3, 69.4 ± 3.6, 72.2 ± 5.7, and 62.5 ± 6.7%, resp.) indicating that administration of excessive propofol has no protective effects. The results did not show significant dose-dependent manner compared with previous reports. This discrepancy may be due to different experimental condition (e.g., the concentration of oxygen in hypoxic chamber, the H/R time and different cell lines that we used).

### 2.2. Inhibitory Effect of Posthypoxia Treatment with Propofol on Apoptosis and Autophagy Induced by H/R

To confirm the inhibitory effect of P-PostH on cell apoptosis, HUVECs were stained with Annexin V-FITC and propidium iodide, and then analyzed by flow cytometry. The percentage of early apoptosis in the control and H/R groups was 8 + 4.4% and 36.6 + 2.4%, respectively (*P* < 0.01 versus control). There was an obvious reduction in the number of early apoptotic cells, (25.6 + 2.8, 22.6 + 2.6, 21 + 3.2, 32.2 + 2.5%) when cells were treated with different concentrations of propofol (25, 50, 100, 150 *μ*mol/L) respectively, compared with the H/R group (*P* < 0.05 versus H/R group) in Figure 2(c). We then examined the apoptosis-related proteins by using Western blotting (WB). Apoptotic protein Bax is upregulated and PARP is cleaved while anti-apoptotic protein Bcl-2 is down-regulated upon H/R treatment (Figure 2(d)). However, the expression of these proteins is reversed and PARP cleavage is prevented upon adding propofol after hypoxia treatment in a dose-dependent manner except 150 *μ*mol/L, which is in agreement with the results from the viability assay and Annexin V-FITC and PI staining (Figure 2(d)).

**Figure 3:**
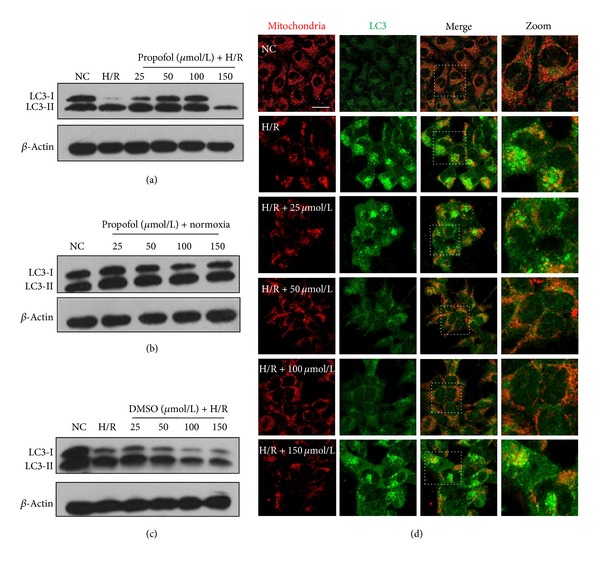
Propofol suppresses the H/R-induced autophagy. (a) The expression of LC3 was determined in normal cells, H/R injury cells, and propofol posthypoxia treatment cells (25–150 *μ*mol/L). (b) The expression of LC3 was determined in control cells and cells treated by normoxia with propofol (25–150 *μ*mol/L). (c) The expression of LC3 was determined in normal cells group, H/R injury cells group, and DMSO posthypoxia treatment groups (25–150 *μ*mol/L). Data are representative WB from 3 independent experiments (*n* = 3). (d) Autophagosomes and mitochondria were probed by anti-LC3 and anti-Tim23 in normal cells, H/R injury cells, and propofol posthypoxia treated cells. Bar, 20 *μ*m.

Next, we investigated whether H/R-induced autophagy in HUVECs can be regulated by propofol. H/R promoted effective transition of LC3-I to LC3-II, which was prevented by propofol treatment except 150 *μ*mol/L, compared with control cells subjected to DMSO during the course of H/R injury or normoxia (Figures 3(a), 3(b), and 3(c)). The immunofluorescence observation strengthened the WB results. HR induced a significant number of LC3 puncta and irregular shape of mitochondria (indicative apoptosis) while cells can be recovered to normal state upon propofol treatment (Figure 3(d)).

Together, these data indicate that Propofol could effectively suppress the apoptosis and autophagy induced by the H/R in HUVECs.

### 2.3. miRNA Are Differently Expressed in P-Post+H/R Compared with H/R Alone

**Figure 4:**
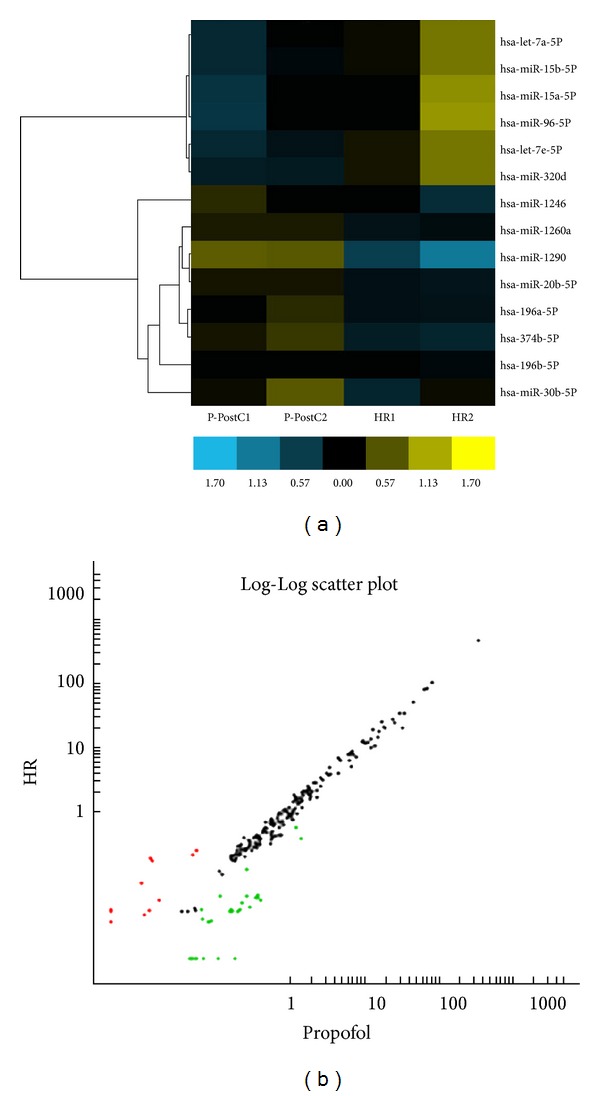
miRNA profiles differentiate the propofol group from the H/R group. (a) Hierarchical clustering of 14 miRNAs whose expression was significantly altered (fold change >2, *P* < 0.01, FDR > 0.05) in the propofol and HR groups. The color stands for the intensity of the signal (*n* = 3). (b) In the scatter diagram, red spots stand for the miRNAs expressed higher in the HR group, the green ones stand for the ones higher in the propofol groups, and the black ones with no significant changes.

**Figure 5:**
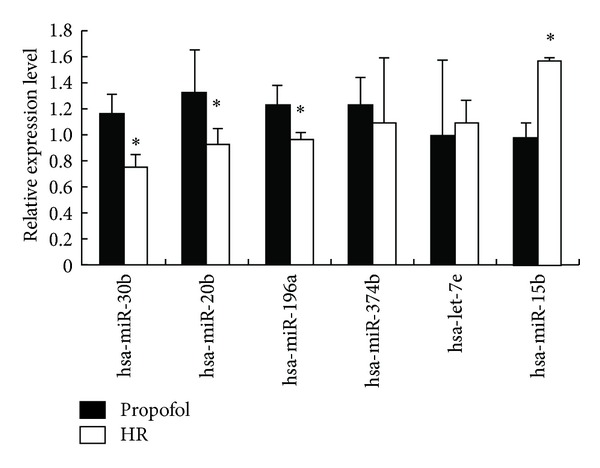
Validation of selected microarray data by qRT-PCR. Statistically significant difference between propofol and H/R is indicated by *P < 0.05 (*n* = 4).

To study the potential miRNAs that may function in the protective effect on H/R injury in HUVECs, we determined the miRNA expression profile in HUVECs through miRNA microarray analysis. We first assessed the miRNA expression profiles in the P-PostH and H/R groups. The expression profiles of hundreds of miRNAs determined to be regulated between P-Post and H/R separate samples into biologically interpretable groups. Among these, 8 miRNAs were identified to be upregulated more than two fold in P-PostH group compared with the same 8 miRNAs in H/R group, while 6 miRNAs were downregulated more than twofold (*P* < 0.01) (Figure 4). 6 miRNAs among these filtered ones were selected for qRT-PCR verification, four of which were validated to be significantly different between the propofol and HR groups (*P* < 0.05). The other two show the expression tendency consistent with the array result but without statistical significance. As is shows in Figure 5, the levels of hsa-miR-30b, hsa-miR-20b, hsa-miR-196a, hsa-miR-374b were upregulated in propofol-treated group with the HR only group, while hsa-let-7e and hsa-miR-15b showed an opposite expression pattern, which was in agreement with the result of microarray hybridization.

### 2.4. Potential Autophagy-Related Targets of the Differentially Expressed miRNAs Are Revealed

**Figure 6:**
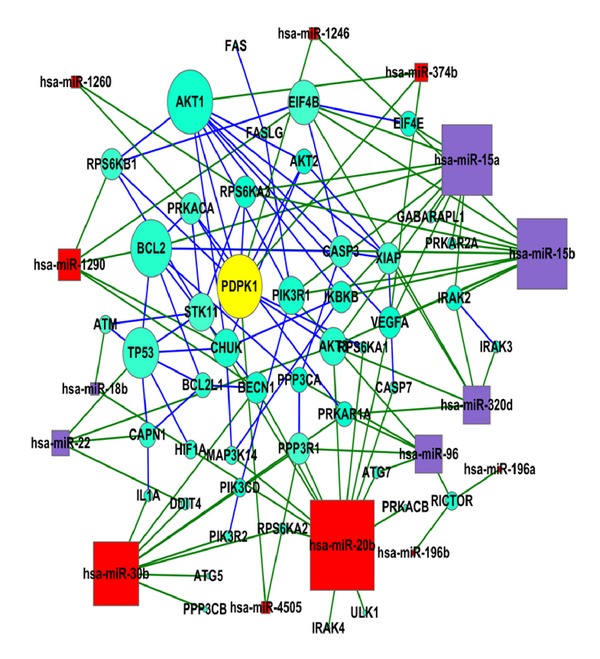
miRNA-gene interaction networks of autophagy and mTOR pathway. Red box nodes represent miRNA that are upregulated in propofol group. Blue represent downregulated ones and green cycle nodes represent mRNA. Edges show the inhibitory effect of miRNA on mRNA.

Computational algorithms showed that hsa-miR-20b, hsa-miR-30b, hsa-miR-96, and hsa-miR-196a may target autophagy-associated genes ULK1, Becline-1, ATG7 and Rictor respectively. Hsa-miR-15b or hsa-miR-374b may target anti-apoptosis- or proliferation-related genes. Hsa-miR-7e may target mitochondria-associated gene (Table 1). All of the reciprocally expressed miRNAs and proteins were predicted by two of the algorithms (Table 1). MTOR inhibition is directly involved in autophagy induction [18, 19]. We found that Hsa-miR-20b, hsa-miR-196 and hsa-miR-15b might be the key regulators of autophagy, mTOR and anti-apoptosis pathway in the protective effects of propofol on H/R injury, respectively. The miRNA-target gene interaction networks related to the autophagy and mTOR pathway which we predicted previously are shown in Figure 6. The upregulated miRNAs hsa-miR-20b showed the 10 target mRNAs when autophagy pathway is concerned, while hsa-miR-30b is also importantly related to autophagy pathway. Hsa-miR-96 and hsa-miR-196a were predicted to target mTOR pathway related genes (Figure 6).

## 3. Discussion

H/R-associated cell injury may involve oxidative stress-induced damage, through formation of reactive oxygen species (ROS). As potent oxidizing and reducing agents, ROS directly damage the endothelium cellular membrane through lipid peroxidation and reductase inhibition [20]. ROS also upregulate the expression of cell adhesion molecules, induce transcription of cytokines, and subsequently stimulate the activation and chemotaxis of neutrophils, which can lead to the death of the endothelial cells [21].

Propofol is structurally similar to the endogenous antioxidant vitamin E and exhibits antioxidant activities [22]. It has a protective effect against oxidative stress-mediated cell injuries [23]. Propofol prevents reactive oxygen species (ROS) such as hydrogen peroxide (H_2_O_2_)-induced cellular damages in cultured endothelial cells and cardiac cells *in vitro* [24, 25] and in hearts with ischemia/reperfusion injury *in vivo* [10].

To date, only a few studies concerning regulation of propofol-mediated cellular protection by microRNA have been reported. Large scale and systemic profiling of key node microRNAs in H/R treated with P-PostH have not been conducted. Consistent with previous results, our study also found the protective effect of propofol against the H/R injury in HUVECs. In order to reveal critical microRNAs involved in this process, we firstly evaluated the miRNA expression profile in the propofol-postconditioned H/R injury of HUVEC cells to reveal the potential role of miRNAs in the protective effect. We found a set of differently expressed miRNAs, with 6 downregulated and 8 upregulated miRNAs in Propofol-postconditioned groups when compared to H/R groups. qRT-PCR of hsa-miR-30b, hsa-miR-20b, hsa-miR-15b, hsa-let-7e, hsa-miR-374b, and hsa-miR-196a further validated the reliability of the microarray result.

In order to gain insight into the function of miRNAs, GO term and KEGG pathway annotation were applied to their target gene pool. KEGG annotation showed a significant change with the autophagy and mTOR signaling pathway in the P-PostH group compared with the H/R group. Further investigation of the miRNA-gene network of these two pathways shows that hsa-miR-20, hsa-miR-30b, and hsa-miR-196a might be the key regulators of autophagy and mTOR pathway, respectively.

Autophagy is an evolutionary conserved process involved in degradation of long-lived or damaged proteins and organelles [26–28]. A previous study showed that propofol protects the autophagic cell death induced by the myocardial I/R injury [14]. Beclin-1 and ATG5 were also shown to be target genes of hsa-miR-30b [29], which were confirmed by our study. Further experiments are needed to probe the detailed mechanism. We also revealed other differentially expressed miRNAs in P-PostH when compared with H/R group which may regulate the MAPK signaling pathway and apoptosis, such as hsa-miR-20b and hsa-miR-15b [30–33].

In conclusion, our study, for the first time, systematically studied the microRNA profiling in P-PostH-treated H/R HUVEC cells and revealed some miRNAs that differentially expressed in P-PostH H/R induced cell injury. Their regulatory roles in the autophagy and anti-apoptosis pathways may be involved in the protective effects of the propofol against H/R injury. The low expression of hsa-miR-30b and hsa-miR-20b may lead to the abnormal upregulation of autophagy-related proteins, induce excessive autophagy, and contribute to the cell death. Confirmation and elucidation of this mechanism requires further study. Present results also point to several exciting directions for future research. Each possible miRNA-gene pair we identified is a strong candidate for a major study to definitively confirm the presence of specific miRNA-gene interactions, thus creating a more detailed picture of the effects of propofol.

## 4. Materials and Methods

### 4.1. Reagents and Antibodies

Propofol was purchased from Sigma Chemical to exclude the influence of lipid emulsion. Regents include Dulbecco's modified Eagle's medium (DMEM), newborn calf serum (NCS), penicillin, streptomycin, trypsin-EDTA (GIBCO Laboratories, Grand Island, New York, USA), dimethylsulfoxide (DMSO), Annexin V-FITC apoptosis detection kit, and CCK-8 cell counting kit. Propofol was dissolved in DMSO and further diluted in phosphate buffered saline (PBS). The final DMSO concentration was 0.1%, which did not affect either cell function or the assay system. The following primary antibodies were used in this study: anti-beta-Actin antibody (Santa Cruz, sc-47778), anti-LC3B polyclonal antibody (Sigma, L7543), anti-LC3 polyclonal antibody (MBL, PM036), anti-TOM20 (FL-145) (Santa Cruz, sc-11415).

### 4.2. HUVEC Cell Culture

HUVECs were isolated from fresh human umbilical cord veins by collagenase digestion according to a modified technique described by Jaffe et al. [34]. Briefly, HUVECs were cultured at 37°C in a 95% O_2_ and 5% CO_2_ humidified atmosphere in DMEM supplemented with 20% fetal bovine serum, 100 *μ*g/mL streptomycin, and 100 IU/mL penicillin. The HUVECs were subcultured nine to ten days later.

### 4.3. Construction of an *In Vitro* Cellular Propofol Posthypoxia Treatment H/R Model

For hypoxia, the culture media was replaced by glucose and serum free DMEM balanced in normal incubator in 30 minutes and the HUVEC cells were then placed in hypoxic conditions which were created by a small enclosed humidified plexiglass chamber filled with 94% N_2_, 5% CO_2_, and 1% O_2_ at 37°C for 12 h. After hypoxia, the medium was washed off, and the HUVECs were returned to the maintenance medium (NCS-DMEM) in normal incubator with 5% CO_2_ and 95% N_2_ for 4 h. At the same time, prepared propofol was added to the medium to different concentrations (25 *μ*mol/L–150 *μ*mol/L). Clinically relevant blood concentration of propofol is approximately 17–62 *μ*mol/L for the maintenance of satisfactory anaesthesia [11]. The blood concentration of bolus injection of propofol can reach 56 *μ*mol/L. Therefore, we consider 25–100 *μ*mol/L as the range of clinically relevant concentrations and increasing propofol concentrations were designed as 25, 50, 100, and 150 *μ*mol/L, respectively (Figure 1).

### 4.4. Cell Viability Assay

The cellular viability was evaluated using a CCK-8 kit. Cells were plated at 1∗10^4^ cells per well in 96-well plates and treated with H/R and various concentrations of propofol posthypoxia treatment. Each concentration of propofol (0–150 *μ*mol/L) was repeated in 6 wells. Meanwhile, increasing concentrations of propofol were given to non-H/R cells for 4 h. After the radiation exposure, the cells were washed twice with PBS and incubated with 1 mL culture medium, which contained 10% CCK-8 solution, for 2 h at 37°C. The absorbance was measured by a multimode microplate reader at 450 nm. The cellular viability (%) was calculated using the formula [(As − Ab)/(Ac − Ab)]∗100%. As: the absorbance of the well containing supernatant from exposure or sham-exposure dishes; Ac: the absorbance of the well containing supernatant from the normal control; Ab: the absorbance of the well containing culture medium with 10% CCK-8 solution.

### 4.5. Detection of Apoptosis

To quantify apoptosis, HUVECs were stained with Annexin V-FITC and propidium iodide. Prepared cells were washed twice with cold PBS and resuspended in 500 mL binding buffer. Five microlitres of Annexin V-FITC and 5 mL of propidium iodide (1 mg/mL) were then added to these cells, which were analysed with a FACSCalibur flow cytometer. Early apoptotic cells were positive for Annexin V and negative for propidium iodide, whereas late apoptotic dead cells displayed both high Annexin V and propidium iodide labelling.

### 4.6. Western Blotting

The expressions of apoptosis related proteins and LC3 were determined by Western blot analysis. The whole cell lysates were prepared in RIPA buffer (50 mM Tris HCl, pH 8, 150 mM NaCl, 1% Nonidet P-40, 0.1% SDS, and 1% Triton X-100 plus proteinase inhibitors; Sigma). Protein concentration was determined by Bradford assay, and samples containing 30 ug were separated by 12% SDS-PAGE. Proteins were transferred to nitrocellulose membranes. After membranes were blocked in 5% nonfat milk in 20 mM Tris-HCl, 150 mM NaCl, and 0.05% Tween 20 for 1 hr at room temperature, the membranes were incubated overnight at 4°C with different primary monoclonal antibodies; *β*-actin antibody was used as a loading control. The membranes were then incubated with secondary antibody conjugated to horseradish peroxide for 1 hr at room temperature and were exposed to enhanced chemiluminescence reagents. Densitometric analysis was performed to quantify the signal intensity.

### 4.7. Immunofluorescence

Cells were grown to 70% confluence on a coverslip. After treatment, cells were washed twice with PBS (Shanghai Sangon Biotech) and fixed with freshly prepared 4% paraformaldehyde at 37°C for 15 min. Antigen accessibility was increased by treatment with 0.1% Triton X-100 (Shanghai Sangon Biotech). After blocking with 1% BSA, cells were incubated with primary antibodies for 1 h at room temperature, and, after washing with PBS, stained with a secondary antibody for a further 50 min at room temperature. Cell images were captured with a TCS SPF5 II Leica confocal microscope.

### 4.8. RNA Extraction and miRNA Microarray Analysis

Total RNA containing small RNA was extracted from H/R groups and P-PostH groups in HUVECs by using the Trizol reagent (Invitrogen) and purified with mirVana miRNA Isolation Kit (Ambion, Austin, TX, USA) according to manufacture's protocol. The purity and concentration of RNA were determined from OD260/280 readings using spectrophotometer (NanoDrop ND-1000). RNA integrity was determined by 1% formaldehyde denaturing gel electrophoresis. The miRNA profiling was performed using Agilent miRNA array. The Agilent array was designed with eight identical arrays per slide (8 × 60 K format), with each array containing probes interrogating 1887 human mature miRNAs and 121 human virus related miRNAs, both from miRBase R18.0. Each miRNA was detected by probes repeat for 30 times.

Microarray experiments were conducted according to the manufacturer's instructions. Briefly, the miRNAs were labeled using the Agilent miRNA labeling reagent. Total RNA (100 ng) was dephosphorylated and ligated with pCp-Cy3, the labeled RNA was purified and hybridized to miRNA arrays. Images were scanned with the Agilent microarray scanner (Agilent), gridded, and analyzed using Agilent feature extraction software version 10.10.

### 4.9. Validation of Selected Microarray Data by qRT-PCR

The differentially expressed miRNAs between H/R and P-PostH levels were determined by qRT-PCR as described [35, 36]. Briefly, RNAs from HUVECs were isolated with mirVana miRNA Isolation Kit (Ambion). QRT-PCR for miRNAs was performed on cDNA generated from 50 ng of total RNA using the protocol of the mirVana quantitative real-time polymerase chain reaction miRNA detection kit (Ambion). As an internal control, U6 was used for miRNAs template normalization. Fluorescent signals were normalized to an internal reference, and the threshold cycle (Ct) was set within the exponential phase of the polymerase chain reaction. The relative gene expression was calculated by comparing cycle times for each target polymerase chain reaction. The target polymerase chain reaction Ct values were normalized by subtracting the U6 Ct value, which provided the ΔCt value. The relative expression level between treatments was then calculated using the following equation: relative gene using the ΔΔCt method with normalization to U6 rRNA endogenous control. The primers used for RT-PCR are shown in Table 2.

### 4.10. Target Gene Prediction

Prediction of miRNA target prediction can be performed by computational algorithms due to their base-pairing rules between miRNA and mRNA target sites, location of binding sequences within the target's 3′UTR, and conservation of target binding sequences within related genomes. In our study, genes that were predicted by TargetScan v5.1 (http://www.targetscan.org/) and Miranda v5 (http://www.mirbase.org/) were regarded as potential targets of a certain miRNA.

### 4.11. Bioinformatic Analysis of Differentially Expressed miRNAs

Gene Ontology (GO) analysis was applied in order to organize genes into hierarchical categories and uncover the miRNA gene regulatory network on the basis of biological process, cellular component, and molecular function. We divided the differentially expressed miRNAs into two groups (H/R upregulated and H/R downregulated) and mapped these two groups to each node of the GO database. The miRNAs corresponding to every node were counted by GSEABase package on the R statistic platform (http://www.r-project.org/). We also analyzed the potential target gene related pathways using GenMAPP v2.1 based on Kyoto Encyclopedia of Genes and Genomes (KEGG) pathway database. The enrichment *P*-value of the target genes involved in every pathway was calculated. Afterward, we integrated the regulatory interactions between the genes and miRNAs. We analyzed two different interactions simultaneously: (1) data from KEGG database describing the relationship between genes, including enzyme-enzyme relation, protein-protein interaction, and gene expression interaction (KEGGSOAP software package (http://www.bioconductor.org/packages/2.4/bioc/html/KEGGSOAP.html)) and (2) protein-protein interactions verified by high-flux experiments (the MIPS mammalian protein-protein interaction database: http://mips.helmholtz-muenchen.de/proj/ppi/). Then, we integrated the results into the gene network, and displayed the figure with the software Medusa 21 (data not shown). Finally, we built certain pathway-related networks using predicted targets for the miRNAs to identify critical miRNAs that might modulate the pathways according to the miRNA degree.

## Figures and Tables

**Table 1:** The targets and the expression levels of microRNAs.

miRNA	Target symbol	Target common name	Miranda	TargetScan	HR expression level
					miRNA
hsa-miR-30b	BECN1	Beclin 1, autophagy related	Yes	Yes	↓
hsa-miR-30b	ATG5	Autophagy related 5	Yes	Yes	↓
hsa-miR-20b	ULK1	unc-51 like autophagy activating kinase 1	Yes	Yes	↓
hsa-miR-20b	MAP3K14	mitogen-activated protein kinase kinase kinase 14	Yes	Yes	↓
hsa-miR-196a	RICTOR	RPTOR independent companion of MTOR	Yes	Yes	↓
hsa-miR-96	ATG7	Autophagy related 7	Yes	Yes	↑
hsa-miR-15b	BCL2	B-cell CLL/lymphoma 2	Yes	Yes	↑
hsa-miR-374b	VEGFA	Vascular endothelial growth factor A	Yes	Yes	↓
hsa-let-7e	ATP2A2	ATPase, Ca++ transporting, cardiac muscle, and slow twitch 2	Yes	Yes	↑

**Table 2:** Primers used in qRT-PCR.

Gene	Annealing temperature °C	Number of gene primer (5′→3′)	Accession number
U6	60	F: 5′CTCGCTTCGGCAGCACATATACT3′	NR_004394.1
		R: 5′CGAATTTGCGTGTCATCCTTGCG3′	
hsa-miR-30b	60	F: 5′CGCTGTAAACATCCTACACTCA3′	MIMAT0000420
		R: 5′GCAGGGTCCGAGGTATTC3′	
Hsa-miR-15b-5P	60	F: 5′ATGGTTCGTGGGTAGCAGCACATCATGGTTTACA3′	MIMAT0000417
		R: 5′GCAGGGTCCGAGGTATTC3′	
Has-miR-196a-5p	60	F: 5′ATGGTTCGTGGGTAGGTAGTTTCATGTTGTTGG3′	MIMAT0000226
		R: 5′GCAGGGTCCGAGGTATTC3′	
Hsa-miR-374b-5P	60	F: 5′CGTGGGATATAATACAACCTGCTAAGTG3′	MIMAT0004955
		R: 5′CTCAACTGGTGTCGTGGA3′	
Hsa-miR-20b-5P	60	F: 5′ATGGTTCGTGGGCAAAGTGCTCATAGTGCAGGTAG3′	MIMAT0001413
		R: 5′CTCAACTGGTGTCGTGGA3′	
Hsa-let-7e	60	F: 5′CGCTGAGGTAGGAGGTTGTA3′	MIMAT0000066
		R: 5′GCAGGGTCCGAGGTATTC3′	

F: forward primer; R: reverse primer.

miRNA number and sequence of a specific miRNA can be obtained from miRBase sequence.
